# Unexpected change in hydrogel spacer volume during external-beam radiation therapy

**DOI:** 10.1007/s11604-024-01617-0

**Published:** 2024-06-26

**Authors:** Naoya Ishibashi, Masaharu Hata, Atsushi Fujikawa, Takao Mochizuki, Toshiya Maebayashi, Masahiro Okada

**Affiliations:** 1 Department of Radiology, Nihon University School of Medicine, 30-1 Oyaguchi Kami-cho, Itabashi, Tokyo 173-8610 Japan; 2 Department of Radiation Oncology, Yokohama City University Graduate School of Medicine, 3-9 Fukuura Kanazawa-ku, Yokohama, Kanagawa 236-0004 Japan; 3 Department of Urology, Yokosuka City Hospital, 1-3-2 Nagasaka, Yokosuka, Kanagawa 240-0195 Japan; 4 Department of Radiology, Yokosuka City Hospital, 1-3-2 Nagasaka, Yokosuka, Kanagawa 240-0195 Japan; 5 Department of Radiology, Nihon University Hospital, 1-6 Kandasurugadai, Chiyoda-ku, Tokyo, 101-8309 Japan

**Keywords:** Hydrogel spacer, Volume increase, External-beam radiation therapy, Prostate cancer

## Abstract

**Purpose:**

To reduce the rectal radiation dose during local radiation therapy of prostate cancer, a hydrogel spacer is typically implanted between the prostate and rectum. However, the spacer volume can change during external beam radiation therapy (EBRT). Therefore, we used magnetic resonance imaging (MRI) to determine changes in the spacer volume during EBRT and analyzed the data to identify patient factors influencing this change.

**Materials and methods:**

A hydrogel spacer was implanted in each enrolled patient diagnosed with prostate cancer (*n* = 22, age = 69–86 years) for EBRT with a total dose of 70 Gy over 35 fractions. T2-weighted MRI images were acquired before (median = 8 days) and during EBRT, when the radiation dose of 48 Gy (median) was given at 55 days (median) after implantation. MRI images were used to determine the spacer volume as well as the maximum and minimum distances between the prostate and anterior wall of the rectum at the middle height of the prostate. Scatterplots were created to determine whether correlations existed between changes in the spacer volume and these two distances, while uni- and multivariate analyses were conducted to determine if the spacer volume change was influenced by the following patient factors: age, body mass index, estimated glomerular filtration rate, and visceral fat areas at the umbilical and femoral head positions.

**Results:**

The spacer volume increased in all 22 patients, with the smaller spacer volume before EBRT increasing by a larger amount during EBRT. This increase in the spacer volume was unaffected by other patient factors. However, it correlated with the change in the maximum distance between the prostate and anterior wall of the rectum.

**Conclusion:**

To avoid adverse changes in the rectal radiation dose during EBRT, hydrogel spacer volume should be monitored, especially if the pre-EBRT volume is small.

## Introduction

Transperineal implantation of a hydrogel spacer, namely SpaceOAR (Boston Scientific Corporation, Marlborough, MA, USA), between the prostate and rectum immediately before local radiation therapy (RT) of prostate cancer has recently been reported to reduce the rectal radiation dose [1–3]. This technique has been reported to reduce radiation-induced toxicity of the gastrointestinal system after RT, irrespective of the RT type, such as external-beam RT (EBRT), brachytherapy, and their combination. A controlled randomized trial focused on EBRT also demonstrated that the hydrogel spacer arm resulted in a significant reduction in the 3-year rectal toxicity [4, 5]. On the basis of these studies, SpaceOAR was listed on the National Health Insurance Drug Price List of Japan in 2018, with increasing numbers of institutions implanting hydrogel spacers. Hydrogel spacers are made from polyethylene glycol (PEG). They maintain the space for 3 months following implantation, after which they begin to degrade through hydrolysis and are completely absorbed within 6 to 12 months for excretion through the kidney in urine [6]. For this reason, monitoring the distance between the prostate and rectum during RT is desirable. Previous studies have variously reported an increase and decrease in the hydrogel spacer volume during EBRT. Because these findings are derived from a small set of cases, there are many unclear aspects regarding the stability of the volume [7–13]. Moreover, there have been no studies on how the hydrogel spacer volume changes in different patient factors. In this study, we measured the hydrogel spacer volume using images from magnetic resonance imaging (MRI) performed immediately before and during EBRT. Subsequently, we examined the relationship between the hydrogel spacer volume and various patient factors, namely the body mass index (BMI), estimated glomerular filtration rate (eGFR), visceral fat area (VFA), and hydrogel spacer volume before EBRT.

## Materials and methods

### Patient characteristics

This study was approved by the institutional review board of Yokosuka City Hospital (No. 2023-9). Between May 2022 and August 2023, 22 patients with prostate cancer were implanted with a hydrogel spacer (SpaceOAR, Boston Scientific Corporation, Marlborough, MA, USA) for EBRT. The patient age was defined as the age at the time of hydrogel spacer implantation. All patients received combined androgen blockade therapy before hydrogel spacer implantation. The obesity parameter, BMI, was calculated from the weight and height at the time of hydrogel spacer implantation. eGFR was estimated using the following conversion formula for Japanese men: eGFR (mL/min/1.73 m^2^) = 194 × serum creatinine (SCr)^−1.094^ × age^−0.287^ [14] before hydrogel spacer implantation. VFA around the implanted hydrogel spacer was evaluated at the umbilical position, as previously described [15], and also at the femoral head position using computed tomography (CT) images obtained for RT planning. These calculations were performed using Ziostation2 version 2.9.x (Ziosoft, Tokyo, Japan).

### Measurement of the hydrogel spacer volume

MRI was performed before and during EBRT. Before EBRT, MRI was performed 8 days (median, range of 7 to 14 days) after hydrogel spacer implantation. During EBRT, MRI was performed on the day when the radiation dose of 48 Gy (median, range of 44 to 58 Gy) was delivered at 55 days (median, range of 49 to 64 days) after implantation. All patients underwent prostate MRI on 3.0 Tesla MR scanners (Skyra, Siemens, Erlangen, Germany) with a body coil. The main parameters of axial T2-weighted images (T2WI) and internal validation set were as follows: the echo time (TE) was 90 ms, the repetition time (TR) was 4000 ms, the spacing between slices was 0.7 mm, the slice thickness was 3.5 mm, the field of view was 220 mm × 220 mm and the voxel size was 0.4 × 0.4 × 3.5 mm^3^. T2WI were acquired because they have been shown to reveal the hydrogel spacer clearly [16]. MRI data were transferred as digital imaging and communications in medicine (DICOM) files to a radiation treatment planning system (Monaco version 5/6, Elekta AB, Stockholm, Sweden) to contour the hydrogel spacer and determine the spacer volume. All contours were defined jointly by the two radiation oncologists (radiation oncologist A, with experience of 20 years and radiation oncologist B, with experience of 30 years) to exclude inter-observer variability. MRI images were also used to measure the maximum and minimum distances between the prostate and anterior wall of the rectum at the middle height of the prostate before and during EBRT [10].

### Radiation therapy

The clinical target volume (CTV) was defined as the prostate ± proximal seminal vesicles, and the planning target volume (PTV) of the CTV had a margin of 8 mm, with 2 mm on the posterior rectal side. EBRT was planned so that 95% of the prescribed dose covered the PTV or at least the entire CTV. All patients were treated with three-dimensional conformal radiation therapy (3D-CRT) consisting of seven tangential beams, with a prescribed dose of 70 Gy over 35 fractions. For only one patient with pelvic lymph node metastasis, irradiation started with the prophylactic pelvic irradiation field, and the boost consisted of seven tangential beams, which was delivered to the PTV of the prostate. Patients did not exhibit acute toxicity related to RT, namely grades of  ≥ 3 according to National Cancer Institute Common Terminology Criteria for Adverse Events 5.0 [17]. Grade 2 urinary retention was observed in only one patient, in whom the hydrogel spacer partially migrated into the prostate, resulting in termination of EBRT after a total radiation dose of 64 Gy.

### Statistical analysis

Data were statistically analyzed using SPSS version 21.0 (IBM, Armonk, NY, USA). Univariate analysis with Pearson’s χ^2^ test and multivariate analysis using logistic regression via the forced entry procedure were performed to evaluate whether patient factors (i.e., age, BMI, eGFR, VFA, and hydrogel spacer volume before EBRT) were associated with spacer volume changes. *P*-values < 0.05 were considered statistically significant. In addition, scattergrams were plotted to correlate the change in the spacer volume with the change in the distance between the prostate and anterior wall of the rectum. Correlation coefficients were calculated using Spearman’s rank correlation.

## Results

**Fig. 1:**
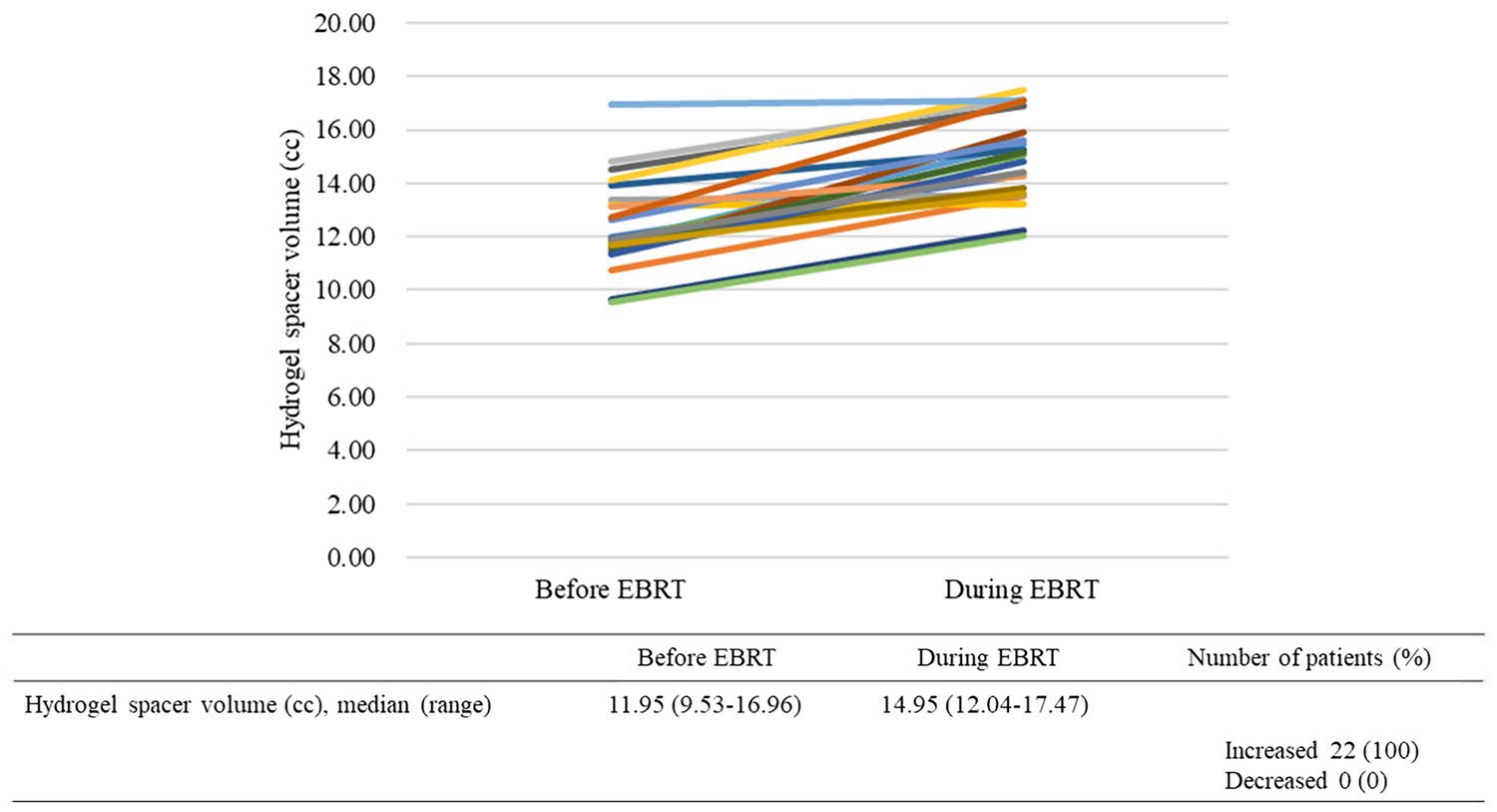
Increase in hydrogel spacer volume during external-beam radiation therapy

**Fig. 2:**
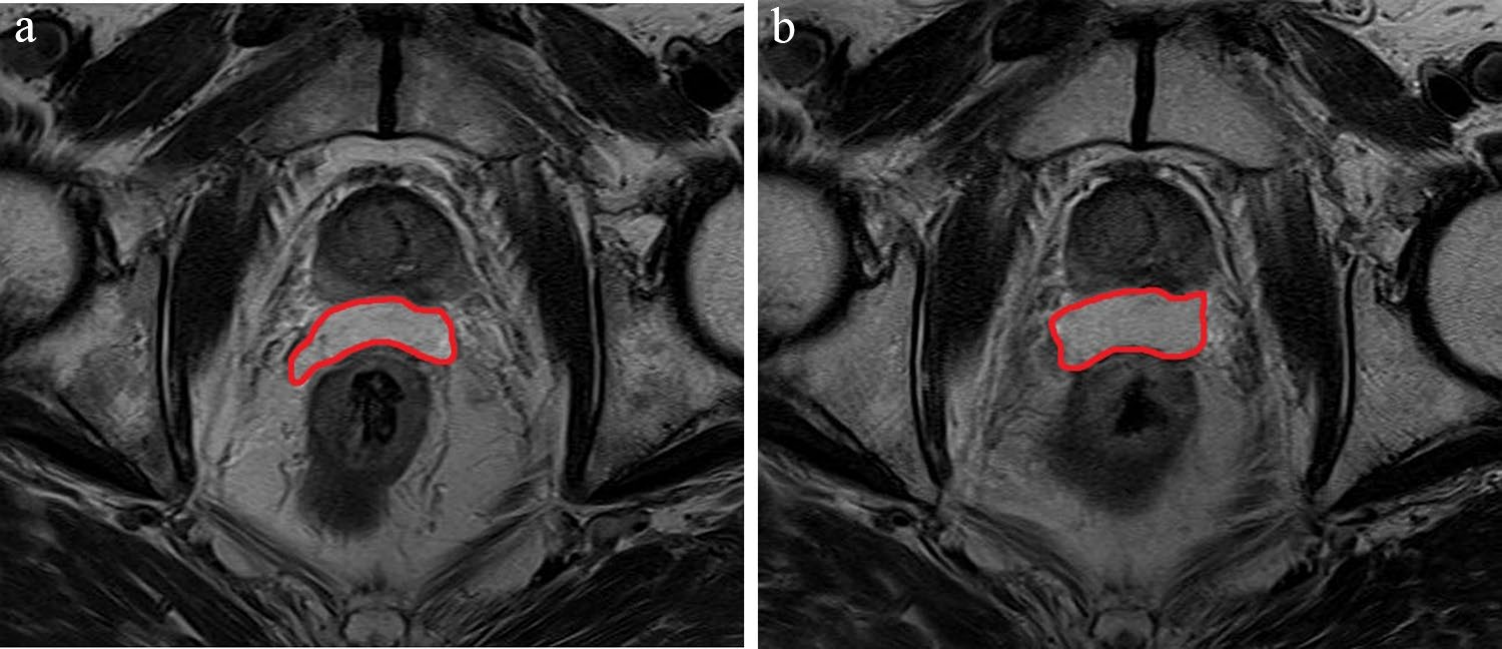
The hydrogel spacer contoured T2-weighted magnetic resonance images before (**a**) and during EBRT (**b**) from the same patient. The spacer volume obviously increased from 12.74 cc to 17.10 cc

**Fig. 3:**
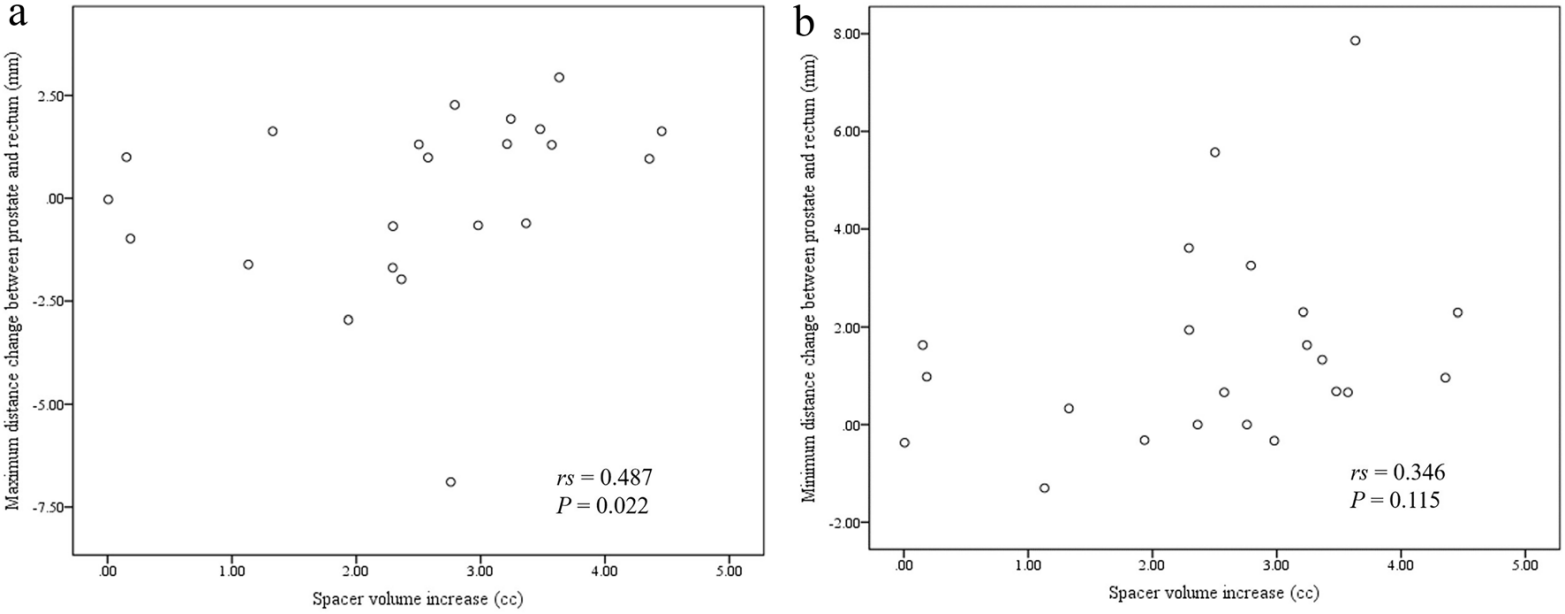
Scattergram of changes in the hydrogel spacer volume and distance between the prostate and anterior wall of the rectum. The change in the spacer volume correlated with the change in the maximum distance (**a**) (*rs* = 0.487 and *P* = 0.022) but not with the change in the minimum distance (**b**) (*rs* = 0.346 and *P* = 0.115)

The characteristics of the 22 patients included in this study are summarized in Table 1. The median age was 80 years (range = 69 to 86 years), while the median BMI was 23.0 kg/m^2^ (range = 17.7 to 27.7 kg/m^2^), and the median eGFR was 55.7 mL/min/1.73 m^2^ (range = 32.5 to 87.8 mL/min/1.73 m^2^). The median VFA at the umbilical position was 94.36 cm^2^ (range = 4.65 to 232.69 cm^2^) and at the femoral head position was 57.93 cm^2^ (range = 5.17 to 94.93 cm^2^). Individual differences in the VFA were large. The median hydrogel spacer volume before EBRT was 11.95 cc (range = 9.53–16.96 cc) and during EBRT was 14.95 cc (range = 12.04–17.47 cc). The hydrogel spacer volume increased during EBRT in all 22 patients (100%) (Fig. 1). The hydrogel spacer contoured MRI images before and during EBRT from the representative patient were shown in Fig. 2. Univariate analysis revealed that the smaller the hydrogel spacer volume before EBRT, the larger the increase in spacer volume during EBRT (*P* = 0.033; Table 2). Multivariate analysis also revealed that the smaller the hydrogel spacer volume before EBRT increased by a larger amount during EBRT (*P* = 0.041; Table 2). No correlation was observed between the spacer-volume increase and other investigated patient factors. The change in the hydrogel spacer volume correlated with the change in the maximum distance between the prostate and anterior wall of the rectum (*rs* = 0.487 and *P* = 0.022) but not with the change in the minimum distance between the prostate and anterior wall of the rectum (*rs* = 0.346 and *P* = 0.115) (Fig. 3).

**Table 1:** Characteristics of patients with prostate cancer (*n* = 22)

Characteristics	Median (range)
Age (years) at hydrogel spacer injection	80 (69–86)
Body mass index (kg/m^2^)	23.0 (17.7–27.7)
eGFR (mL/min/1.73 m^2^)	55.7 (32.5–87.8)
Visceral fat area at the umbilical position (cm^2^)	94.36 (4.65–232.69)
Visceral fat area at the femoral head position (cm^2^)	57.93 (5.17–94.93)
Pretreatment PSA (ng/ml)	11.6 (2.0–166.8)
T stage (no.)	
T1a	1
T1c	2
T2a	4
T2c	6
T3a	6
T3b	3
Gleason score (no.)	
6	2
7	9
8	6
9	5
MRI before EBRT from hydrogel spacer injection (days)	8 (7–14)
MRI during EBRT from hydrogel spacer injection (days)	55 (49–64)

*eGFR* estimated glomerular filtration rate, *PSA* prostatic-specific antigen, *MRI* magnetic resonance imaging, *EBRT* external beam radiation therapy

**Table 2:** Univariate and multivariate analyses of factors that increase the hydrogel spacer volume

Factors	Univariate analysis			Multivariate analysis		
	Odds ratio	95% CI	*P*-value	Odds ratio	95% CI	*P*-Value
Age at hydrogel spacer injection (years)						
< 80/ ≥ 80	0.214	0.035–1.307	0.087	0.060	0.003–1.052	0.054
BMI (kg/m^2^)						
< 23.0/ ≥ 23.0	0.694	0.130–3.720	0.670	0.645	0.043–9.743	0.751
eGFR (mL/min/1.73 m^2^)						
< 55.7/ ≥ 55.7	0.694	0.130–3.720	0.670	1.002	0.086–11.700	0.999
VFA at the umbilical position (cm^2^)						
< 94.36/ ≥ 94.36	1.440	0.269–7.714	0.670	5.429	0.296–99.484	0.254
VFA at the femoral head position (cm^2^)						
< 57.93/ ≥ 57.93	0.694	0.130–3.720	0.670	0.215	0.009–5.288	0.347
Hydrogel spacer volume before EBRT (cc)						
< 11.95/ ≥ 11.95	0.141	0.022–0.918	**0.033**	0.059	0.004–0.891	**0.041**

Values in bold are statistically significant

*BMI* body mass index, *eGFR* estimated glomerular filtration rate, *VFA* visceral fat area, *CI* confidence interval, *EBRT* external beam radiation therapy

## Discussion

Because the hydrogel spacer might decrease in size 1 to 2 weeks after implantation, CT for RT planning is recommended at least 3 to 5 days after implantation [7]. Thereafter, the space created by the hydrogel spacer is maintained for 3 months. According to several reports, if EBRT is completed within this period, then the distance between the prostate and anterior wall of the rectum will not change [3, 6, 18]. However, studies measuring the hydrogel spacer volume have variously reported increases and decreases during EBRT (Table 3). The seven studies that we found in the literature include five studies reporting an increase and two studies reporting a decrease. However, accurate measurements using CT might be difficult because of insufficient contrast between the spacer and surrounding tissues, such as the prostate [7, 8]. Among the studies using MRI images for measurements, most reported an increase in the spacer volume, while only one reported a decrease in the spacer volume. The spacer volume differences between the results of previous studies seem to be largely related to the timing of imaging and the number of days after spacer implantation. The decrease might be attributed to the long period until the second measurement, which is 74 to 93 days after implantation [11]. In all of our cases, the hydrogel spacer volume increased. This increase in the hydrogel spacer volume is attributable to tissue edema at the early stage of RT and changes in the gas volume of the spacer [7, 8]. PEG is used to prepare not only hydrogel spacers but also various medical products. When it is injected into a blood vessel in vivo, PEG is distributed across the kidney and organs of the reticuloendothelial system, such as the liver and spleen [19]. The biokinetics of subcutaneously injected PEG was recently revealed through an in vivo study, which reported that PEG distribution across the kidney and liver after subcutaneous injection depends on its molecular weight [20]. However, the molecular weight of PEG in the hydrogel spacer employed in this study has not been disclosed, and thus its biokinetics remains unknown. Because the PEG hydrogel spacer was injected into perirectal fatty tissues instead of blood vessels, we evaluated VFA around the hydrogel spacer in addition to renal function. Our results revealed that neither renal function nor VFA affected the hydrogel spacer volume. In contrast, the spacer volume increased by a larger amount if it was smaller before EBRT. In addition, because the change in the hydrogel spacer volume was correlated with the change in the maximum distance between the prostate and anterior wall of the rectum. In the previous hydrogel spacer clinical trials, PTV margin was determined variously from 4 to 10 mm to the CTV also in the posterior rectal side [3, 5, 21]. These studies were setting on intensity-modulated radiation therapy and reduced rectal toxicity. Our results revealed that the change in the hydrogel spacer volume correlated not with the change in the minimum distance between the prostate and anterior wall of the rectum. For example, in some of our cases with increasing hydrogel spacer volume, the change in the minimum distance between the prostate and anterior wall of the rectum changed to a minus quantity. Even if the hydrogel spacer volume increased larger during EBRT, the minimum distance between the prostate and anterior wall of the rectum may remain close. Therefore, it is considered better not to expand our PTV margin of 2 mm in the posterior rectal side to the CTV on 3D-CRT setting.

**Table 3:** Studies on changes in the hydrogel spacer volume during radiation therapy

Study	Hydrogel spacer volume	No. of patients	RT type	Measurement modality	Time of first measurement	Volume of first measurement (cc)	Time of second measurement	Volume of second measurement (cc)
Pinkawa et al. [7]	Decreased	3	IMRT	CT	Placement day	30 (mean)	7 to 14 days after placement^a^	10 (mean)
Pinkawa et al. [8]	Increased	15	IMRT	CT	Before RT	9.9 (mean)	Last RT week	11.5 (mean)
Hama et al. [9]	Increased	7	IMRT	MRI	Within 14 days after placement	11.9 (mean)	Mean 49.9 days after placement	14.3 (mean)
Fukumitsu et al. [10]	Increased	30	PBT	MRI	2 to 30 days after placement	11.1	44 to 71 days after placement	12.8
Kubo et al. [11]	Decreased	4	EBRT after BT	MRI	At EBRT planning	14.4 (median)	74 to 93 days after placement	7.1 (median)
Brenneman et al. [12]	Increased Decreased Unchanged	15 4 2	IMRT or PBT	MRI	Placement day	11.0 (median)	20 to 39 days after placement	10.8 (median)
Saito et al. [13]	Increased	15	SBRT	MRI	1 to 9 days after placement	11.9 (mean)	14 to 25 days after first MRI	12.8 (mean)
Our cases	Increased	22	3D-CRT	MRI	7 to 14 days after placement	11.95 (median)	49 to 64 days after placement	14.95 (median)

*RT* radiation therapy, *IMRT* intensity-modulated radiation therapy, *PBT* proton beam therapy, *EBRT* external beam radiation therapy, *3D-CRT* three-dimensional conformal radiation therapy, *BT* brachytherapy, *SBRT* stereotactic body radiation therapy, *CT* computed tomography, *MRI* magnetic resonance imaging

^a^Before radiation therapy

This study has some limitations. First, the patient population was small at a single institution. Second, the rectal dose volume histogram (DVH) could not be evaluated because CT for RT re-planning at MRI during EBRT was not performed. In the future, we have planned to perform RT re-planning CT at MRI during EBRT and evaluate the relation between the increase hydrogel spacer volume during EBRT and rectal DVH in the large patient population.

In this time, we intended to understand the relationship between the hydrogel spacer volume change and various patient factors. It is desirable to carefully monitor the distance between the prostate and rectum during EBRT, especially if the hydrogel spacer volume before EBRT is small.
